# Zapalenie Jelita Cienkiego i Okrężnicy Wywołane Białkami Pokarmowymi (FPIES) – Rzadka Choroba o Częstych Objawach – Kompendium Dla Lekarza Praktyka

**DOI:** 10.34763/devperiodmed.20192301.6778

**Published:** 2019-04-08

**Authors:** Hanna Ludwig, Aneta Krogulska

**Affiliations:** 1 Katedra i Klinika Pediatrii, Alergologii i Gastroenterologii, Collegium Medicum im. Ludwika Rydygiera w Bydgoszczy, Uniwersytet Mikołaja Kopernika w Toruniu, Toruniu Polska

**Keywords:** zapalenie jelita cienkiego i okrężnicy wywołane białkami pokarmowymi, alergia IgE-niezależna, doustna próba prowokacji pokarmowej, dieta eliminacyjna, food protein-induced enterocolitis syndrome, non-lgE-mediated food allergy, oral food challenge, elimination diet

## Abstract

Zapalenie jelita cienkiego i okrężnicy wywołane białkami pokarmowymi (food protein-induced enterocolitis syndrome – FPIES) jest rodzajem lgE-nieza/eżnej alergii pokarmowej, o różnym stopniu ciężkości. Ostra postać choroby manifestuje się wymiotami, nadmierną sennością i bladością skóry, które pojawiają się zwykle w ciągu 7-4 godzin od spożycia pokarmu wyzwalającego, i może powadzić do wstrząsu. Pokarmy indukujące objawy to najczęściej: mleko krowie, soja, ryż i owies. Przewlekła postać FP/ES jest typowa dla niemowląt karmionych mlekiem modyfikowanym lub mieszanką sojową i objawia się nawracającymi wymiotami, biegunką oraz słabymi przyrostami masy ciała. U większości pacjentów z FP/ES, do ustalenia rozpoznania i zidentyfikowania pokarmów wyzwalających objawy wystarcza szczegółowa analiza wywiadu chorobowego. W przypadkach wątpliwych przydatna jest doustna próba prowokacji pokarmowej. Leczenie FP/ES polega na eliminacji pokarmów wywołujących objawy, monitorowaniu postępu choroby oraz edukacji opiekunów. Większość dzieci nabywa tolerancję pokarmową w wieku 3-5 lat

## Abstract (en)

food protein-induced enterocolitis syndrome (FP/ES) is a non-/gE-mediated food allergy with varying degrees of severity. The acute form of the disease is manifested by vomiting, lethargy and pallor, which usually appear within 7-4 hours after food ingestion, and can lead to shock. The most common trigger foods are: cow's milk, soy, rice and oats. Chronic FP/ES is typical for infants fed with cow's milk or soy infant formula and is manifested by chronic vomiting, diarrhea and failure to thrive. In the vast majority of patients with FP/ES, the analysis of the clinical history is sufficient to diagnose and identify trigger foods. If the history is unclear, use an ora/ food challenge to help confirm the diagnosis. Long-term management of patients with FP/ES involves elimination of the trigger foods, monitoring for FP/ES resolution and caregivers' education. The majority of chi/dren acquire food to/erance at the age of 3-5.

## WSTĘP

Wymioty i biegunka należą do częstych objawów w praktyce klinicznej, jednak nie zawsze stanowią przejaw „grypy jelitowej”. Niekiedy mogą być oznaką zapalenia jelita cienkiego i okrężnicy wywołanego białkami pokarmowymi (food protein-induced enterocolitis syndrome – FPIES), **czyli postacią IgE-niezależnej alergii** pokarmowej prowokowanej alergenami pokarmowymi [1, 2, 3, 4, 5, 6, 7, 8]. Pomimo istniejącego potencjalnego zagrożenia życia wynikającego z możliwości ciężkiego przebiegu choroby, w tym wstrząsu, znajomość jej jest niska. Może to wynikać ze stosunkowo rzadkiego występowania, niedoboru dobrej jakości badań epidemiologicznych czy braku jednoznacznie ustalonych kryteriów rozpoznawania i postępowania [2, 4, 8]. W 2017 r. opublikowano raport międzynarodowej grupy ekspertów, którym przewodniczyła prof. Nowak-Węgrzyn A., stanowiący próbę usystematyzowania obecnej wiedzy na temat FPIES [1]. W niniejszym artykule, opierając się głównie na przeglądzie bazy PUBMED z ostatnich 5 lat, omówiono zasady rozpoznawania, diagnostyki i leczenia FPIES, co może ułatwić lekarzom praktykom postępowanie z chorym dzieckiem już na etapie podstawowej opieki zdrowotnej.

## Epidemiologia

Choć pierwszy opis FPIES pochodzi z 1940 r., to schorzenie po raz pierwszy zostało formalnie zdefiniowane w połowie lat siedemdziesiątych ubiegłego wieku [3]. Wg Międzynarodowej Klasyfikacji Statystycznej Chorób i Problemów Zdrowotnych kod odpowiadający FPIES to K52.2 [2]. Dane na temat częstości występowania FPIES są ograniczone i zróżnicowane [1, 3]. Na podstawie prospektywnego badania kohorty urodzeniowej Katz i wsp. określili częstość występowania FPIES na 0,34%, tj. 3/1000 noworodków/2 lata; wszyscy pacjenci rozwinęli FPIES w ciągu pierwszych 6 miesięcy życia [9]. W badaniu populacji dzieci w wieku poniżej 2 lat z Australii, stwierdzono, że szacowana częstość występowania FPIES wynosi 15,4/100 000/rok [10].

**Choroba ujawnia się zwykle między 2 a 7 mż**., przy czym FPIES indukowany białkami mleka krowiego (BMK) czy soją dotyczy zwykle niemowląt <6 mż., a indukowany pokarmami stałymi – niemowląt między 6 a 12 mż., zgodnie z czasem wprowadzania pokarmów do diety [1, 3]. FPIES występuje głównie u niemowląt karmionych sztucznie, zazwyczaj 1-4 tygodnie po wprowadzeniu mieszanki mlecznej, natomiast zasadniczo nie występuje u dzieci karmionych piersią (bądź opisy są incydentalne). Schorzenie może rozwinąć się także u osób dorosłych [7].

## Obraz kliniczny

FPIES należy „traktować, jako potencjalny stan nagły, stanowiący zagrożenie życia, objawiający się wymiotami o opóźnionym początku i/lub wodnistą/krwistą biegunką, które w ostateczności mogą spowodować niestabilność hemodynamiczną oraz spadek ciśnienia tętniczego krwi, u co najmniej 15% pacjentów” [1]. Objawy i ciężkość przebiegu FPIES zależą od dawki i częstotliwości spożywania „szkodliwego” pokarmu, jak również od wieku wystąpienia pierwszych objawów. Choroba może zaczynać się **wcześnie**, tj. poniżej 9 mż. lub **późno**, tj. powyżej 9 mż. [11]. Przebieg może być ciężki bądź łagodny. Dla **łagodnej** postaci choroby typowe są: nawracające wymioty z/lub bez biegunki, bladość i niewielka senność. **W ciężkiej** postaci występują: nawracające, nasilone wymioty z/lub bez biegunki, bladość, senność, odwodnienie, hipotensja, wstrząs i zaburzenia metaboliczne. W zależności od przebiegu wyróżnia się ostrą i przewlekłą postać choroby [1, 2, 8, 12].

FPIES - postać ostra występuje, gdy pokarm wyzwalający jest spożywany w pewnych odstępach czasu lub po okresie jego wcześniejszej eliminacji. Charakterystyczne dla tej postaci są nawracające wymioty, rozpoczynające się w ciągu 1-4 godzin od przyjęcia pokarmu, z towarzyszącą nadmierną sennością i bladością skóry. Niekiedy występuje wodnista biegunka (czasem z krwią lub śluzem), która pojawia się zwykle w ciągu 5-10 godzin od spożycia pokarmu, najczęściej w ciągu doby. Objawy zwykle ustępują w ciągu 24 godzin. Rozwój dziecka jest zazwyczaj prawidłowy, a pomiędzy epizodami ostrego FPIES nie obserwuje się żadnych niepokojących objawów. Opóźniony początek oraz brak objawów skórnych i oddechowych sugerują reakcję ogólnoustrojową, ale odmienną od anafilaksji. Ciężkie reakcje mogą prowadzić do hipotermii, methemoglobinemii, rozwoju kwasicy metabolicznej, niedociśnienia i wstrząsu, co może początkowo sugerować posocznicę [1, 11, 12]. Należy pamiętać, że w przeciwieństwie do zakażeń przewodu pokarmowego czy sepsy, gdzie objawy ustępują wolno (w ciągu dni), ostre reakcje FPIES zwykle całkowicie ustępują w ciągu kilku godzin. Po zastosowaniu diety eliminacyjnej pacjent pozostaje bezobjawowy.

**Kryteria rozpoznania postaci ostrej FPIES**: pacjent powinien spełnić kryterium większe i co najmniej 3 kryteria mniejsze [1].

**Kryterium większe**: wymioty pojawiające się w ciągu 1-4 godzin po spożyciu podejrzanego pokarmu i brak typowych IgE-zależnych alergicznych objawów skórnych lub oddechowych.

**Kryteria mniejsze**:

1. Drugi (lub kolejny) epizod nawracających wymiotów po spożyciu podejrzanego pokarmu.
2. Nawracające wymioty w ciągu 1-4 h po spożyciu pokarmu.
3. Nadmierna senność.
4. Nadmierna bladość skóry.
5. Konieczność wizyty w oddziale ratunkowym.
6. Konieczność zastosowania płynoterapii dożylnej.
7. Biegunka w ciągu 24 godzin (zwykle 5-10 godzin od spożycia podejrzanego pokarmu).
8. Hipotensja.
9. Hipotermia.

**FPIES – postać przewlekła** rozwija się u niemowląt karmionych regularnie mieszanką mleczną lub sojową. Obj awia się okresowymi wymiotami, przewlekłą biegunką, zaburzeniami przyrostu masy ciała i rozwoju fizycznego. Ciężkie postaci przewlekłego FPIES mogą także prowadzić do odwodnienia i wstrząsu. Po wyeliminowaniu szkodliwego pokarmu objawy ustępują w ciągu 3-10 dni [13]. Ponowne spożycie w wyniku przypadkowej lub kontrolowanej ekspozycji, np. podczas doustnej próby prowokacji (OFC), indukuje ostrą reakcję w ciągu 1-4 godzin od spożycia. Nagłe wystąpienie objawów, po okresie eliminacji szkodliwego pokarmu, odróżnia tę postać FPIES od innych przewlekłych schorzeń gastroenterologicznych o podobnym przebiegu [1, 8, 11, 14].

**Kryteria rozpoznania postaci przewlekłej FPIES**: brak jednoznacznych kryteriów większych i mniejszych, ale określono objawy typowe dla łagodnej i ciężkiej postaci przewlekłego FPIES [1,8].

**Postać łagodna –** pokarm indukujący objawy spożywany jest w niższych dawkach (np. alergeny pokarmowe w mleku matki, pokarmy stałe). Objawy: okresowe wymioty i/lub biegunka, zwykle słabe przyrosty masy ciała (< 10 g/dzień u młodych niemowląt*), ale bez cech odwodnienia i kwasicy metabolicznej [1, 8, 12].

**Postać ciężka –** pokarm indukujący objawy występuje regularnie w diecie chorego (np. mleko modyfikowane). Objawy: okresowo występujące, intensywne wymioty, biegunka (również z krwią), czasami odwodnienie i kwasica metaboliczna [8, 12].

**Najważniejszym kryterium rozpoznania przewlekłego FPIES jest ustąpienie objawów w ciągu kilku dni po wyeliminowaniu szkodliwego pokarmu(ów) z diety i nagły nawrót objawów po ich ponownym spożyciu**. Bez potwierdzenia dodatnim wynikiem OFC, rozpoznanie przewlekłego FPIES pozostaje domniemane.

### Czynniki wpływające na obraz kliniczny FPIES

Na obraz kliniczny FPIES mają wpływ takie czynniki jak: **wiek pacjenta, miejsce zamieszkania** czy **współ-występowanie IgE-zależnej alergii na pokarmy** (IgE-zależna AP).

Niemowlęta z FPIES wywołanym przez białka mleka krowiego (BMK) lub soję w wieku poniżej 2 miesięcy, wykazują istotnie większe ryzyko wystąpienia biegunki, krwi w kale i opóźnienia rozwoju fizycznego, w porównaniu ze starszymi niemowlętami, które częściej prezentują same wymioty, bez biegunek [1].

Istnienie różnic narodowościowych w obrazie klinicznym FPIES sugerują wyniki badań dzieci o różnym miejscu zamieszkania. Nomura i wsp. zaobserwowali występowanie gorączki u 13% oraz krwawych stolców u 47% badanych niemowląt z Japonii, a aż 47% dzieci miało dodatnie wyniki specyficznych przeciwciał IgE (Sie) [16]. Ten fenotyp choroby wydaje się odmienny od opisów z krajów zachodnich, gdzie dominują wymioty, a współwystępowanie dodatnich slgE i/lub punktowych testów skórnych (PTS) waha się od 3% w Australii do 24-25% w USA [3, 17, 18]. Wg Caubet i wsp., w USA najczęstszymi objawami choroby były wymioty (96% chorych) oraz hipotensja (19%), a najrzadziej biegunka (7%) [19]. W badaniu z Izraela kolejność objawów była

następująca: wymioty – 100%, nadmierna senność – 77%, biegunka – 25% i biegunka z krwią – 4,5%, a dodatni wynik PTS odnotowano jedynie u 5% chorych [9]. Przewlekła postać FPIES występuje rzadko, ale wydaje się częściej rozpoznawana w Japonii i Korei, niż w innych krajach [1, 3].

## Patofizjologia

Patofizjologia FPIES nie jest do końca poznana. Uważa się, że główną rolę w rozwoju procesu zapalnego w jelicie pełnią antygenowo swoiste limfocyty T, które w wyniku aktywacji przez alergeny pokarmowe uwalniają cytokiny prozapalne [1]. Wzrost czynnika martwicy guza (TNFa) oraz zmniejszona ekspresja receptorów dla transformującego czynnika wzrostu β (TGF-β) w błonie śluzowej jelit, działających protekcyjnie wobec bariery nabłonkowej, skutkuje zwiększeniem przepuszczalności jelitowej. Mechanizmy odpowiedzi humoralnej w przebiegu FPIES są słabo scharakteryzowane. Dotychczas wykazano zwiększone wydzielanie przeciwciał IgA, IL-2, IL-5, IL-8, IL-9, a zmniejszone wydzielanie IL- 10, związanej z rozwojem tolerancji pokarmowej u pacjentów z FPIES [20, 21]. Doniesienia o skutecznym zastosowaniu ondansetronu w leczeniu objawowym u pacjentów z FPIES sugeruje dodatkowe zaangażowanie mechanizmów neuroimmunologicznych w etiopatogenezie choroby [22, 23].

## Diagnostyka i rozpoznanie

Dla FPIES nie ma charakterystycznych badań, a rozpoznanie opiera się przede wszystkim na wywiadzie, w którym stwierdza się występowanie powtarzalnych, charakterystycznych objawów po spożyciu określonego pokarmu, z poprawą po jego eliminacji [1,6]. Jeśli wywiad jest typowy, próba prowokacji pokarmowej (oral food challenge – OFC) nie jest konieczna. Jeśli dane z wywiadu są niepewne, po wykluczeniu innych potencjalnych przyczyn występowania dolegliwości ze strony przewodu pokarmowego w danym wieku, dla potwierdzenia podejrzenia FPIES przydatne jest przeprowadzenie OFC. Warto podkreślić, że u większości pacjentów z FPIES o ostrym przebiegu, sama analiza wywiadu chorobowego wystarcza do ustalenia rozpoznania i zidentyfikowania pokarmów wyzwalających, a OFC wykorzystywana jest głównie do monitorowania rozwoju tolerancji pokarmowej [1, 6, 24].

Wykonanie OFC w celach diagnostycznych powinno być zarezerwowane w przypadku, gdy [1, 5, 24]:

- wywiad chorobowy jest niejasny,
- pokarm wyzwalający jest niezidentyfikowany,
- przebieg objawów jest nietypowy, np. objawy pojawiają się po kilku minutach od spożycia przy braku slgE lub dolegliwości utrzymują się pomimo eliminacji podejrzanego pokarmu z diety,
- podejrzewa się przewlekły FPIES (z uwagi na mniej specyficzny charakter objawów i trudniejszą identyfikację pokarmów wyzwalających).

**Protokół wykonania OFC**:

Każda OFC wymaga ścisłego nadzoru lekarza. Przed próbą zaleca się zabezpieczenie obwodowego dostę-pu dożylnego (50% dzieci z dodatnim wynikiem OFC może wymagać leczenia płynami dożylnymi) [1, 2, 6]. **Dawkę pokarmu prowokującego określono jako 0,06 do 0,6 g białka/kg masy ciała (zwykle 0,3 g białka na kg masy ciała)** [1, 6, 24]. Tę dawkę należy podać w 3 równych częściach, co 10-15 minut, tj. w ciągu 30-45 minut. Następnie należy obserwować pacjenta przez 4-6 godzin. Nie zaleca się przekroczenia łącznej dawki 3 g białka lub 10 g całkowitego pokarmu (100 ml płynu) do wstępnego karmienia, które ma na celu przybliżenie do spożycia przez dziecko całkowitej wielkości porcji [1,6, 24]. W przypadku braku reakcji w ciągu 4 godzin, podaje się drugie karmienie, zazwyczaj pełną porcję pokarmu (stosowną do wieku), z następową 4-godzinną obserwacją [6, 24]. U pacjentów, u których w przeszłości obserwowano ciężkie reakcje po spożyciu pokarmu, można rozważyć zastosowanie niższych dawek początkowych oraz wydłużyć okres obserwacji pomiędzy dawkami [1, 6, 24]. Wynik OFC należy uznać za dodatni, jeśli zostanie spełnione kryterium większe i co najmniej 2 kryteria mniejsze [1,8].

**Kryterium większe dodatniej OFC**: wymioty pojawiające się w ciągu 1-4 godzin po spożyciu podejrzanego pokarmu i brak klasycznych IgE-zależnych alergicznych objawów skórnych lub oddechowych.

**Kryteria mniejsze dodatniej OFC**:

- Nadmierna senność
- Bladość
- Biegunka w ciągu 5-10 godzin po spożyciu pokarmu
- Hipotonia
- Hipotermia
- Zwiększona liczba neutrofilów o ponad 1500 komórek/ ml krwi w stosunku do liczby wyjściowej (wzrost ten osiąga maksimum po 6 godzinach od spożycia pokarmu spustowego).

Przyjmuje się, że lekarz prowadzący może uznać wynik OFC za dodatni, nawet, jeśli zostało spełnione tylko kryterium większe, bez żadnych kryteriów mniejszych, ponieważ nie zawsze istnieje możliwość oceny liczby neutrofilów w odpowiednim czasie. W przypadku ujemnego wyniku OFC zaleca się regularne spożywanie pokarmu [8].

## Różnicowanie

Rozpoznanie FPIES nie jest łatwe i często bywa opóźnione [11, 14, 17]. Jak wykazał Mehr i wsp. prawidłową diagnozę ustalono tylko u 11% pacjentów z ostrym FPIES [17]. Pierwsze epizody choroby mogą być błędnie interpretowane jako „grypa jelitowa”, nieżyt żołądkowojelitowy lub sepsa, szczególnie jeśli przebiegają ze złym stanem ogólnym, hipotensją, kwasicą metaboliczną czy leukocytozą z przesunięciem w lewo. Szczególnie trudno ustalić rozpoznanie w przypadku przewlekłego FPIES, wymagającego przeprowadzenia szerokiej diagnostyki różnicowej [1, 7, 11]. Różnicowanie ostrej i przewlekłej postaci FPIES przedstawiono w tabeli I i tabeli II. Chociaż nie są znane testy laboratoryjne typowe dla rozpoznania FPIES, istnieje wiele diagnostycznych badań pomocniczych, za pomocą których można wykluczyć inne jednostki chorobowe, o podobnej do FPIES symptomatologii.

**Tabela I:** Diagnostyka różnicowa ostrego FPIES [1, 12, 14]. Table I. Differential diagnosis of acute FPIES [1, 12, 14].

Jednostki chorobowe *Disease entity*		Cechy różnicujące *Differentiating features*
Infekcyjne *linfections*	Posocznica *Sepsis*	Gorączka lub temperatura <36°C, niekiedy wybroczyny na skórze, wysokie wykładniki stanu zapalnego, dodatnie wyniki badań bakteriologicznych, sama resuscytacja płynowa jest nieefektywna, objawy ustępują wolno *Fever or body temperature <36°C, sometimes petechiae at physical examination, inflammatory indices are elevated, positive results of bacteriological tests, fluid resuscitation alone not effective, symptoms resolve slowly*
	Zakażenie przewodu pokarmowego *Infection of digestive tract*	Pojedynczy epizod choroby, kontakt z chorym zakaźnie w wywiadzie, gorączka, zwykle występuje biegunka (we FPIES czasami brak biegunki), badania mikrobiologiczne stolca (na obecność wirusów i/lub bakterii) są pozytywne *Single episode of illness, contacts with infectious disease, usually fever and diarrhea (In FPIES there is no fever, sometimes no diarrhea), stool studies for viruses and bacteria are positive*
Chirurgiczne *Surgical*	Martwicze zapalenie jelit *Necrotizing enterocolitis (NEC)*	Dotyczy głównie noworodków i młodszych niemowląt, obecność czynników ryzyka w wywiadzie (wcześniactwo, niedotlenienie okołoporodowe, niska masa urodzeniowa, niewydolność oddechowa, wrodzone wady serca), krwiste stolce, tkliwość powłok brzusznych, zaburzenia krzepnięcia, pneumatosis intestinalis w badaniach radiologicznych, sama resuscytacja płynowa jest nieefektywna *Affects newborns and younger infants, risk factors:prematurity, perinatal hypoxia, low birth weight, respiratory distress, congenital heart disease; bloody stools, coagulation disorders, pneumatosis intestinalis on abdominal radiographs, fluid resuscitation alone not effective (required antibiotic therapy, parenteral nutrition, surgical treatment)*
	Pylorostenoza *Pyloric stenosis*	Objaw „oliwki" w badaniu przedmiotowym, w badaniach laboratoryjnych zasadowica hipochloremiczna, cechy przerostowego zwężenia odźwiernika w USG, leczenie chirurgiczne *Olive-shaped mass in the baby's abdomen at physical examination, hypochloremic alkalosis, features of hypertrophic pyloric stenosis on ultrasound, surgical treatment*
	Wgłobienie *Intussusception*	Napadowe, krótkotrwałe bóle brzucha, objaw „galaretki malinowej", wyczuwalny guz w jamie brzusznej w badaniu przedmiotowym, objaw tarczy strzelniczej w USG *Periodic, cramping abdominal pain, „red currant jelly" stool, a palpable abdominal mass at physical examination, target sign on abdominal ultrasound*
Alergiczne *Allergic*	Anafilaksja *Anaphylaxis*	Symptomy rozpoczynają się zwykle nagle, w ciągu 1-15 minut od ekspozycji na alergen (rzadziej po >1 godzinie, jak w FPIES), dodatkowo występują objawy IgE-zależne (pokrzywka, świąd skóry, kaszel, obrzęk naczynioruchowy), może postępować szybko i prowadzić do zatrzymania oddechu, drgawek i utraty przytomności w ciągu 1-2 minut, w badaniach dodatkowych pozytywne wyniki slgE *Symptoms usually begin suddenly, within 1-15 minutes of exposure to the alllergen (less requently after 1 hour, as in FPIES), usually other IgE-mediated symptoms (hives, itching, cough, angioedema), reaction may progress rapidly and leads to cessation of breathing, seizures, and loss of consciousness within 1-2 min., positive slgE results*

**Tabela II:** Diagnostyka różnicowa przewlekłego FPIES [1, 12, 14]. Table II. Differential diagnosis of chronic FPIES [1, 12, 14].

Jednostki chorobowe *Disease entity*		Cechy różnicujące *Differentiating features*
Gastroenterologiczne *Gastroenterological*	Zapalenie prostnicy i odbytnicy wywołane przez białka pokarmowe *Food protein-induced allergic proctocolitis (FPIAP)*	Dotyczy głównie niemowląt karmionych wyłącznie piersią, typowym objawem jest oddawanie stolców z niewielką ilością świeżej krwi, dobry stan ogólny dziecka i zwykle prawidłowy rozwój, brak wymiotów *Mainly in infants exclusively breastfed, blood-streaked stools are a typical symptom, infant is in good general condition and typically thriving, no vomiting*
	Refluks żołądkowo-jelitowy *Gastroesophageal reflux disease (GERD)*	Objawy tylko z górnego odcinka przewodu pokarmowego, wymioty o przewlekłym charakterze i łagodniejszym przebiegu (zwykle nie doprowadzają do odwodnienia), brak związku ze spożyciem konkretnych pokarmów *Symptoms only from the upper gastrointestinal tract, vomiting more chronic and milder than in FPIES (usually does not lead to dehydration), no relation to specific food intake*
	Eozynofilowe zapalenie przełyku, eozynofilowe zapalenie żołądka i jelit *Eosinophilic esophagitis (EoE) or eosinophilic gastroenteritis*	U niemowląt - trudności w karmieniu, odmowa przyjmowania pokarmów, u starszych dzieci – dysfagia oraz epizody uwięźnięcia kęsów pokarmowych, wymioty o łagodniejszym przebiegu, częściej dodatnie wyniki badań slgE oraz kilka pokarmów wyzwalających objawy *In infants -feeding difficulties, food intake refusal, in older children – dysphagia and episodes of getting food stuck in the esophagus; vomiting less severe; more often positive IgE test results and multiple food triggers*
	Celiakia *Coeliac disease*	Objawy związane ze spożywaniem glutenu, manifestacja pozajelitowa choroby (objawy neurologiczne, hematologiczne, metaboliczne), dodatnie markery serologiczne celiakii, współwystępowanie innych chorób autoimmunologicznych *Symptoms associated with the consumption of gluten, parenteral manifestation (neurological, hematological, metabolic), results of celiac serology are positive, coexistence of other autoimmune diseases*
	Nietolerancja laktozy - hypolaktazja *Lactose intolerance (hypolactasia)*	Wzdęcia, skurczowe bóle brzucha, biegunka, nudności oraz wymioty (rzadziej) po spożyciu mleka lub produktów mlecznych zawierających laktozę; brak objawów po spożyciu produktów mlecznych poddanych fermentacji *Abdominal bloating, cramps, diarrhea, nausea and (less often) vomiting after consumption of milk or dairy products containing lactose; no symptoms after ingestion of fermented dairy products*
	Enteropatie o podłożu immunologicznym: NZJ, wtórne niedobory odporności *Immune bowel disease, enteropathies: secondary inflammatory immunodeficiencies*	Rzadko u niemowląt, brak związku ze spożyciem konkretnych pokarmów *Rare in infancy, no relation to specific food intake*
Chirurgiczne *Surgical*	Choroba Hirschsprunga *Hirschsprung disease*	Opóźnienie pasażu smółki, powiększony obwód brzucha, niechęć do ssania, zaparcia stolca, epizody niedrożności przewodu pokarmowego *Delayed passage of meconium, enlarged abdominal circumference, reluctance to suck, constipation, gastrointestinal obstruction episodes*
	Zespoły niedrożności jelit (pasma Ladda, niedokonany zwrot jelit) *Gastrointestinal obstruction (Ladd bands, intestinal malrotation)*	Brak związku ze spożyciem konkretnych pokarmów, objawy niedrożności w badaniach radiologicznych *No relation to specific food intake, evidence of obstruction on radiologic studies*
Metaboliczne *Metabolic*	Defekty cyklu mocznikowego, acydemie organiczne, zaburzenia β-oksydacji kwasów tłuszczowych, nietolerancja fruktozy *Urea cycle defects, organie acydemia, β-oxidation defects, fructose intolerance*	Opóźnienie rozwoju psychoruchowego, objawy neurologiczne, powiększenie narządów wewnętrznych, dodatnie wyniki specyficznych testów laboratoryjnych surowicy i moczu *Developmental delay, neurological symptoms, organomegaly, positive results of specific laboratory tests of serum and urine*
Inne *Other*	Zaburzenia neurologiczne *Neurologic disorders*	Brak związku ze spożyciem konkretnych pokarmów, objawy neurologiczne *No relation to specific food intake, neurological symptoms*
	Pierwotne niedobory odporności *Primary immunodeficiencies*	Brak związku ze spożyciem konkretnych pokarmów, częste infekcje jelitowe *No relation to specific food intake, frequent gastrointestinal infections*
	Zaburzenia układu krzepnięcia *Coagulation defects*	Brak związku ze spożyciem konkretnych pokarmów *No relation to specific food intake*
	Niedobór alfa 1-antytrypsyny α*1-Antitrypsin deficiency*	Brak związku ze spożyciem konkretnych pokarmów, objawy uszkodzenia wątroby *No relation to specific food intake, hepatic involvement*
	Zaburzenia psychospołeczne (Zespół Münchausena by proxy, awersja pokarmowa) *Psychosocial disorders (Münchhausen syndrome by proxy, food aversion)*	Pomocna analiza psychologiczna *Psychological analysis is helpful*

## Alergeny pokarmowe a FPIES

U 83% pacjentów, FPIES jest wywoływany przez 1 alergen, u 17% przez 2 alergeny a u 5-10% przez 3 alergeny [16, 25]. Do najistotniejszych pokarmów wyzwalających FPIES należą **mleko krowie** i **soja**. Alergia na te pokarmy często współistnieje ze sobą. W badaniach Ruffner i wsp., u około połowy pacjentów z FPIES indukowanym mlekiem krowim, obserwowano reakcję na soję [25]. Podobne dane przedstawiają inni badacze z USA [18, 26, 27, 28]. Jednakże w Australii, Korei, Izraelu, Hiszpanii i Włoszech zjawisko to obserwowano rzadko lub wcale [3, 9,17, 29, 30].

FPIES może rozwinąć się wskutek reakcji na pokarmy uzupełniające takie jak: **ziarna zbóż** (ryż, owies, pszenica, jęczmień, kukurydza), kurczak, indyk, wołowina, ryby (szczególnie w Hiszpanii i Włoszech), orzechy, warzywa (słodkie i białe ziemniaki, fasolka szparagowa, pomidor, dynia), rośliny strączkowe: orzeszki ziemne, zielony groszek, soczewica; owoce (jabłka, gruszki, banany, brzoskwinie) i owoce morza [1, 3, 4, 17]. Jajo kurze także może być pokarmem wywołującym FPIES, ale rzadko. Opóźnione rozpoznawanie FPIES na pokarmy uzupełniające może wynikać z faktu, iż ryż, owies i warzywa rzadko są postrzegane, jako pokarmy alergizujące. U ok. 65% dzieci z FPIES na 1 pokarm uzupełniający współistnieje FPIES na mleko i/lub soję, a u 80% rozwija się reakcja na drugi pokarm stały [27]. Dziecko z FPIES wywołanym przez jeden gatunek zbóż, ma 50% ryzyko rozwoju reakcji na inne ziarna, choć nie odnotowano reakcji na mąkę pszenną u dzieci z FPIES indukowanym przez ryż czy owies.

Dane na temat współwystępowania FPIES na kilka różnych pokarmów są zróżnicowane. W USA odnotowywano najwięcej przypadków reakcji na 2 lub 3 pokarmy, natomiast w Australii, a przede wszystkim we Włoszech, wyraźnie dominuje „1-pokarmowe” FPIES [3]. Na zróżnicowanie geograficzne może nakładać się wiele czynników, takich jak: nawyki żywieniowe danej narodowości, rozpowszechnienie karmienia piersią, częstość stosowania mieszanek sojowych, obecność chorób atopowych w danej populacji czy determinacja genetyczna [1, 2, 3].

## Współwystępowanie chorób atopowych

Pomimo tego, iż FPIES jest postacią IgE-niezależnej alergii pokarmowej (AP), u wielu pacjentów obserwuje się współwystępowanie chorób atopowych. Rodzinne obciążenie atopią dotyczy 54% pacjentów z FPIES [3]. Około 30-50% dzieci z FPIES ma towarzyszące atopowe zapalenie skóry (AZS) [17, 19, 26, 28], a do 15% pacjentów – IgE-zależną AP [18]. Warto podkreślić, że dawka alergenu prowokująca objawy jest większa w przypadku FPIES, niż w IgE-zależnej AP [19]. Astma, alergiczny nieżyt nosa i eozynofilowe zapalenie przełyku rzadziej towarzyszą FPIES, co najprawdopodobniej wynika z rozwoju tych chorób w późniejszym okresie życia [3].

## Badania laboratoryjne w FPIES

U niemowląt z przewlekłym FPIES w badaniach laboratoryjnych obserwuje się różny stopień niedokrwistości, hipoalbuminemii i leukocytozę z eozynofilią. Badanie stolca może ujawnić obecność krwi utajonej, neutrofilów, eozynofilów, kryształów Charcota-Leydena i/lub substancji redukujących [12].

## Endoskopia w FPIES

Nie zaleca się rutynowego wykonywania badań endoskopowych z biopsją [1]. Mogą one jednak być przydatne w diagnostyce różnicowej, celem wykluczenia innych zaburzeń przewodu pokarmowego, szczególnie gdy objawy są ciężkie i nie ustępują mimo wstrzymania karmienia lub zastosowania diety elementarnej [11]. U pacjentów z FPIES, w ocenie histologicznej obserwowane są: nacieki komórek tucznych, limfocytów, eozynofilii, ropnie krypt, a także różnego stopnia zaniki kosmków [12].

## Badania alergologiczne w FPIES

### Swoiste alergenowo IgE

Nie należy rutynowo oceniać slgE dla alergenów pokarmowych u pacjentów z FPIES. U **90% z nich wyniki punktowych testów skórnych (PTS), jak również slgE są ujemne w momencie wstępnej diagnozy** [9, 17, 25, 27, 30]. Oznaczenie slgE, można rozważyć u chorych z współistniejącymi chorobami atopowymi, jak również w trakcie dalszej obserwacji, ponieważ u 2% do 20% dzieci z FPIES mogą pojawić się slgE dla alergenów wyzwalających objawy, a od 20% do 40% na inne powszechne alergeny pokarmowe [1, 18, 19]. Obecność sIgE wykazano u 18-30% dzieci z FPIES wywołanym mlekiem/ soją i u 21% dzieci z FPIES wywołanym pokarmami uzupełniającymi [9, 27]. Biorąc pod uwagę ryzyko konwersji typowego FPIES w postać atypową, z możliwością wystąpienia reakcji IgE-zależnych, u pacjentów z FPIES indukowanym BMK, przed wykonaniem OFC, należy oznaczyć stężenie slgE na mleko [1].

Związek między FPIES a obecnością Sie można tłumaczyć w dwojaki sposób. Zwiększona przepuszczalność jelitowa u chorych z FPIES, może skutkować zwiększoną przenikalnością alergenów pokarmowych, co prowadzi do wytworzenia Sie. Odwrotnie, występujące miejscowo w błonie śluzowej jelit przeciwciała IgE, mogą ułatwiać wychwyt alergenów, a w konsekwencji prowadzić do indukcji „ogólnego” procesu zapalnego jelit.

### Atopowe testy płatkowe z alergenami pokarmowymi (ATP)

Biorąc pod uwagę potencjalny udział alergenowo swoistych limfocytów T, w patofizjologii FPIES oraz w mechanizmie powstawania odczynów podczas wykonywania ATP, metoda ta wydaje się być obiecującym narzędziem diagnostycznym. Istnieją pojedyncze badania, które oceniają przydatność testów w diagnostyce FPIES [28, 31]. Określają one swoistość ATP na poziomie 71-86%. Jednak z powodu sprzecznych danych dotyczących przydatności ATP w identyfikacji pokarmów wyzwalających FPIES, aktualnie nie ma zaleceń co do ich rutynowego wykonywania [1, 25].

## Postępowanie w ostrym epizodzie FPIES

Ostre epizody FPIES mogą przebiegać w sposób łagodny (1-2 epizody wymiotów), umiarkowany (>3 epizodów wymiotów i nadmierna senność) lub ciężki (>3 epizodów wymiotów, senność patologiczna, bladość/ szarość skóry, hipotonia). Ten ostatni może prowadzić do wystąpienia wstrząsu hipowolemicznego. Wówczas należy niezwłocznie założyć obwodowy dostęp dożylny i rozpocząć intensywną resuscytację płynem izotonicznym (np. bolusy soli fizjologicznej w dawce 10-20 ml/kg, powtarzane w razie potrzeby) oraz roztwór dekstrozy w postaci ciągłego wlewu dożylnego (terapia podtrzymująca). Pojedyncza dawka dożylnego metyloprednizolonu (1 mg/kg; maksymalnie 60-80 mg/dawkę), przypuszczalnie może ograniczyć rozwój procesu zapalnego, jednak żadne badania nie rekomendują ich bezwzględnego zastosowania. Należy również monitorować i w razie potrzeby korygować kwasicę metaboliczną i zaburzenia elektrolitowe. W ciężkich reakcjach pacjenci mogą wymagać także tlenoterapii, wentylacji mechanicznej, zastosowania wazopresorów, wodorowęglanów lub błękitu metylenowego w methemoglobinemii. Auto-strzykawki z adrenaliną nie są rutynowo zalecane dla pacjentów z FPIES. Wyjątek stanowią chorzy, z współistniejącą alergią IgE-zależną, którzy w opinii lekarza prowadzącego, są potencjalnie narażeni na wystąpienie anafilaksji [1,5].

**Ryc. 1:**
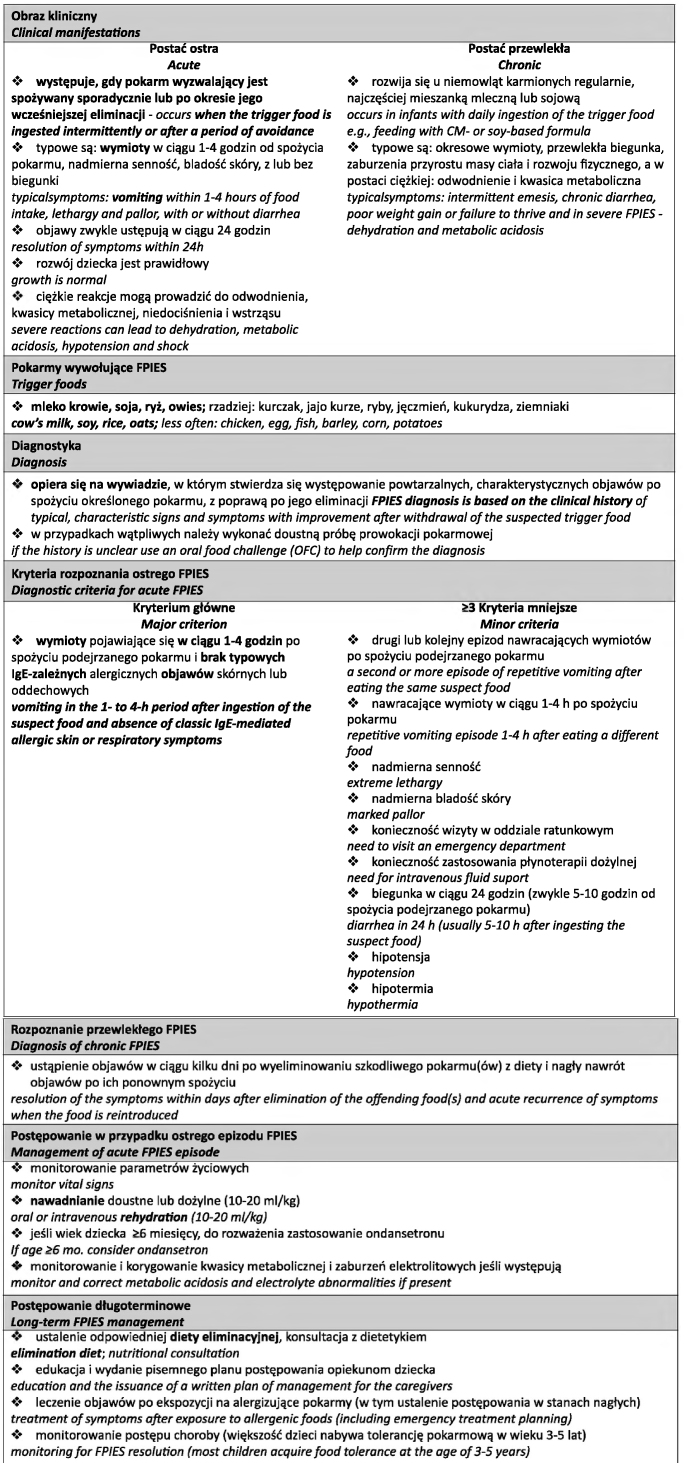
Kompendium podsumowujące aktualny stan wiedzy o FPIES dla lekarzy praktyków. Fig. 1. Compendium summarizing the current state of knowledge about FPIES for practitioners.

Niezależnie od ciężkości objawów ostrego epizodu FPIES, u dzieci >6 miesięcy, można rozważyć zastosowanie ondansetronu (domięśniowo lub dożylnie 0,15 mg/kg/dawkę; maksymalnie 16 mg/dawkę) jako leczenia wspomagającego wymioty [1, 2, 22, 23, 32]. Stosując lek należy zachować ostrożność u dzieci z chorobami serca, z uwagi na możliwość wywołania wydłużenia odstępu QT [22].

Na podstawie danych wskazujących, że większość ostrych epizodów zdarza się w domu, ustępuje bez leczenia, i nie kończy się zgonem, Sopo i wsp. sugerują, że w trakcie ostrego, łagodnego epizodu FPIES u dzieci powyżej 1 r.ż, można przyjąć postawę wyczekującą, ale tylko pod warunkiem możliwego dostępu do natychmiastowego założenia drogi dożylnej i podania sterydów. U dzieci młodszych zawsze wskazane jest natychmiastowe założenie dostępu dożylnego. Ważnym elementem postępowania jest poinformowanie opiekunów o konieczności doustnego nawadniania. Ponadto wskazane jest zaopatrzenie pacjenta w pisemny plan postępowania [8, 32].

## Postępowanie długoterminowe

Do podstawowych zasad postępowania z dzieckiem z FPIES należy:

- **ustalenie odpowiedniej diety eliminacyjnej**
- edukacja opiekunów dziecka
- leczenie objawów po ekspozycji na alergizujące pokarmy (w tym ustalenie postępowania w stanach nagłych)
- monitorowanie postępu choroby, w tym nabywania tolerancji pokarmowej
- przeprowadzenie konsultacji dietetycznej, celem ustalenia zasad przestrzegania diety eliminacyjnej i zastosowania suplementacji zapobiegającej wystąpieniu niedoborów żywieniowych [1, 33].

Niemowlętom z podejrzeniem FPIES wywołanym przez BMK lub soję zaleca się unikanie wszelkich form tych pokarmów, w tym produktów pieczonych, chybaże są one już zawarte w diecie i dobrze tolerowane [1, 5, 32]. Nie ma jeszcze jednoznacznych badań oceniających tolerancję „wypiekanego mleka i jajka” u dzieci z FPIES [34]. Jeśli to możliwe, należy kontynuować karmienie piersią. W przypadku niemowląt karmionych sztucznie, należy zastosować mieszanki mlekozastępcze o wysokim stopniu hydrolizy białek (eHF) lub mieszanki elementarne (AAF). Zastosowania AAF może wymagać ok. 10-40% dzieci z FPIES indukowanym białkami mleka krowiego (BMK) [1, 19].

Z uwagi na ryzyko współwystępowania FPIES na mleko i soję, należy rozważyć nadzór lekarza podczas wprowadzania soi do diety i odwrotnie [1, 18, 25, 26, 27]. Mleko kozie i owcze nie jest zalecane u pacjentów z FPIES indukowanym BMK. Możliwe jest natomiast zastosowanie mleka wielbłądów lub osłów.

## FPIES a karmienie piersią

Większość niemowląt nie reaguje na alergeny pokarmowe obecne w mleku matki, co wynika z faktu trawienia białek pokarmowych przez matkę oraz obecności w mleku matczynym IgA i TGFß. Wg Nowak-Węgrzyn i wsp. u żadnego dziecka karmionego piersią nie obserwowano objawów FPIES na pokarm spożywany przez matkę [27]. Objawy pojawiały się dopiero gdy pokarm indukujący został podany bezpośrednio niemowlęciu. Autorzy zaobserwowali ponadto, że u dzieci karmionych wyłącznie piersią, przyczyną FPIES są pokarmy stałe (zboża, warzy­wa, drób). Inne wnioski wysnuli Monti i wsp. oraz Sopo i wsp. opisując przypadki młodych niemowląt z FPIES wywołanym przez BMK, zawarte w pokarmie matczynym [35, 36]. Japońscy badacze zaobserwowali objawy FPIES wywołane alergenami pokarmowymi zawartymi w mleku matki u 10%, a badacze z Australii u 5% dzieci [10,16]. Wg Jarvinen i Nowak-Węgrzyn dieta eliminacyjna u matki powinna być wdrożona jedynie w przypadku, gdy objawy występują po karmieniu piersią i obserwuje się zaburzenia przyrostu masy ciała u niemowlęcia [8]. Jeśli nie uda się złagodzić objawów za pomocą diety eliminacyjnej u matki, należy rozważyć przerwanie karmienia piersią i wprowadzenie AAF [1, 37].

## Zalecenia żywieniowe

Czas ponownego wprowadzenia wcześniej eliminowanego pokarmu do diety dziecka jest zmienny. Zależy od dotychczasowego przebiegu choroby. Sposób wprowadzenia (w szpitalu czy w domu) powinien być zawsze wspólnie ustalony między lekarzem i opiekunem dziecka, uwzględniając wiek pacjenta, liczbę pokarmów wyzwalających, wyniki slgE, ciężkość poprzednich reakcji FPIES, a także komfort opiekuna oraz dostęp i odległość do lokalnych oddziałów ratunkowych [6].

Dzieci z FPIES indukowanym BMK lub soją mają zwiększone ryzyko reakcji na pokarmy stałe, najczęściej ryż lub owies, jednak **nie zaleca się opóźnionego wprowadzania pokarmów uzupełniających do diety u tych dzieci** [1]. Bazując na doświadczeniu klinicznym ekspertów i opublikowanych raportach dotyczących pokarmów wyzwalających FPIES, sformułowano zasady rozszerzania diety u dzieci z FPIES [1, 33]. Zaleca się rozpoczęcie wprowadzania pokarmów stałych od warzyw z grupy niskiego ryzyka, do których należą: brokuł, kalafior, pasternak, rzepa i dynia. **Słodkie ziemniaki i zielony groszek** zakwalifikowano do grupy **wysokiego ryzyka**, w której znalazły się także: **mleko krowie, soja, płatki owsiane, ryż, drób, jajo kurze, ryby i banany**. Za bezpieczne dla pacjentów z FPIES uznano owoce, takie jak: truskawki, jagody, śliwki, brzoskwinie, arbuz i awokado, wśród pokarmów mięsnych - jagnięcinę i wołowinę, a wśród ziaren - komosę ryżową i proso. Autorzy podkreślają jednak, iż zawsze należy brać pod uwagę preferencje żywieniowe danego dziecka. Wówczas pomocne może być użycie produktów z grupy umiarkowanego ryzyka, które powszechnie występują w diecie polskich niemowląt, jak np. marchewka, biały ziemniak, zielona fasolka, jabłko, gruszka, kaszka pszenna czy chrupki/płatki kukurydziane lub jęczmienne. Według Venter i Groetch wprowadzanie zbóż można rozpocząć od kukurydzy, następnie jęczmienia, owsa i na końcu ryżu [33]. Tolerancja na jeden pokarm z danej grupy produktów żywnościowych jest uważana za korzystny wskaźnik prognostyczny dla tolerancji na inne pokarmy z tej samej grupy [1, 38].

U niemowląt z ciężkim FPIES na BMK i/lub soję zaleca się nadzorowane przez lekarza wprowadzanie pokarmów stałych. Należy je wprowadzać pojedynczo, z następową 4-dniową obserwacją [1, 38]. Z uwagi na narażenie pacjentów z FPIES na ryzyko utraty masy ciała czy spowolnienie wzrastania, parametry rozwoju fizycznego dziecka powinny być oceniane w regularnych odstępach czasu [1, 5].

## Nabywanie tolerancji pokarmowej

Rozwój tolerancji na BMK i soję następuje wcześniej, niż na pokarmy stałe. W badaniu Lee i wsp. z Australii, w wieku 3 lat 88% dzieci tolerowało mleko krowie, a 87% ryż, podczas gdy procent pacjentów tolerujących jaja i ryby wynosił odpowiednio: 12,5% i25% [39]. Autorzy tego badania zaobserwowali ponadto, że **starszy wiek pacjenta w czasie wystąpienia pierwszego epizodu FPIES** czy ustalenia diagnozy oraz **dodatni wynik PTS, wiąże się z wolniejszym nabywaniem tolerancji pokarmowej**. Caubet i wsp. również obserwowali wpływ współwystępowania IgE-zależnej AP na wydłużenie czasu nabywania tolerancji u pacjentów z FPIES [19]. Płeć, współwystępowanie jakiejkolwiek choroby atopowej, liczba pokarmów wyzwalających oraz stopień ciężkości początkowego epizodu FPIES, wg australijskich badaczy nie miały znaczenia rokowniczego [39]. Wyniki różnych badań obrazują różnice narodowościowe w zakresie nabywania tolerancji pokarmowej. Wg badań koreańskich, tolerancja na mleko krowie i soję u pacjentów z FPIES pojawia się przed ukończeniem 2 roku życia (odsetek niemowląt z tolerancją BMK i soi wynosił odpowiednio: 63,6% i 91,7% po 10 miesiącach) [29]. Wg Katz i wsp. z Izraela 60% dzieci z FPIES na mleko nabyło tolerancję w 1 rż, 75% w 2 rż, a 85% w 3 rż. [9]. Średni wiek nabycia tolerancji u amerykańskich pacjentów z niewykrywalnymi poziomami slgE wynosił 5 lat dla mleka i 6,7 dla soi [19]. W brytyjskim badaniu opartym na kwestionariuszu, aż24,7% dzieci w wieku 8 lat, nadal reagowało na niektóre alergeny pokarmowe [40].

Nie ma zaleceń co do konkretnego czasu wykonania OFC celem oceny nabycia tolerancji pokarmowej u pacjentów z FPIES, jednak zazwyczaj są one podejmowane wciągu 12 do 18 miesięcy od ostatniej reakcji [1, 29]. FPIES jest schorzeniem obarczonym możliwościami wystąpienia pomyłek diagnostycznych, narażających dziecko na występowanie kolejnych epizodów choroby bądź ryzyko niepotrzebnego stosowania diet eliminacyjnych, stąd wiedza na ten temat wymaga ciągłej aktualizacji.

## Wnioski

1. FPIES jest postacią IgE-niezależnej alergii pokarmowej o różnym stopniu ciężkości, występującą u niemowląt i małych dzieci.
2. Ostra postać choroby manifestuje się wymiotami, nadmierną sennością i bladością skóry, które pojawiają się zwykle w ciągu 1-4 godzin od spożycia pokarmu, i/lub biegunką 5-10 godzin po ekspozycji.
3. Przewlekła postać FPIES jest typowa dla niemowląt karmionych mlekiem modyfikowanym lub mieszanką sojową i objawia się nawracającymi wymiotami, biegunką oraz zaburzeniami przyrostu masy ciała.
4. Pokarmami, które najczęściej indukują FPIES są: mleko krowie, soja, ryż i owies.
5. Rozpoznanie opiera się na wywiadzie; w wątpliwych przypadkach przydatna jest doustna próba prowokacji pokarmem.
6. Leczenie polega na zastosowaniu diety eliminacyjnej.
7. Większość dzieci nabywa tolerancję pokarmową w wieku 3-5 lat.
